# Breast Reconstruction With Local Flaps: Don't Forget Grandma

**Published:** 2019-12-10

**Authors:** Bradley J. Vivace, Swapnil D. Kachare, Michael Ablavsky, Sara R. Abell, Luke T. Meredith, Christina N. Kapsalis, Joshua H. Choo, Bradon J. Wilhelmi

**Affiliations:** ^a^School of Medicine, University of Louisville, Louisville, KY; ^b^Division of Plastic and Reconstructive Surgery, Department of Surgery, University of Louisville, Louisville, KY

**Keywords:** breast reconstruction, local flaps, oncoplastic surgery, bilobed flap, rhomboid flap

## Abstract

**Objective:** Lateral breast defects of various causes can be reconstructed with random patterned local flaps utilizing oncoplastic techniques. These local flaps are used frequently in other areas but are infrequent in breast reconstruction despite affording excellent utility in small lateral defects. We sought to demonstrate this with a case series involving 5 patients who underwent oncoplastic breast surgery with random patterned flap reconstruction. **Methods:** From 2016 to 2017, 3 different varieties of random flaps were used in 5 women requiring lateral breast defect reconstruction secondary to resection of localized cancer or cutaneous lesion. The local flaps included a rhomboid flap, the bilobed flap, and a rotational flap. Patients were then evaluated in the clinic 10 to 12 months postoperatively for complications, symmetry, and satisfaction of reconstruction. **Results:** In 4 of 5 patients, the local flap remained fully viable and there was no incidence of seroma, infection, or further complications. One patient developed a post-operative hematoma requiring evacuation and a second patient experienced distal flap necrosis and delayed wound healing. Patients reported satisfaction with the reconstruction. **Conclusions:** Several random patterned local flaps exist for a variety of breast defects. They can yield excellent cosmetic results, high patient satisfaction, and bolster a low rate of complications. Our case series emphasizes the utility of random patterned flaps for lateral breast oncoplastic reconstruction.

The random patterned flap (RPF) is infrequently used in oncoplastic breast surgery (OBS).^1^^,^^2^ It is of particular utility in women with ample axillary or peripheral laxity to provide volume for defects located in the superior pole and lateral quadrants.^3^ Kronowitz et al^4^ noted that local tissue rearrangements involving random blood supply of local breast tissue or adjacent axillary tissue resulted in superior cosmetic outcomes and lower rates of complications versus myocutaenous flaps in the setting of immediate reconstruction.

We sought to demonstrate the utility of RPFs in OBS via a case series involving 5 patients who underwent lumpectomy or resection of cutaneous lesions with reconstruction via 3 different iterations of RPFs.

## METHODS

A total of 5 patients underwent local flap reconstruction of lateral breast defects between the years 2016 and 2017 by a single surgeon in the Division of Plastic and Reconstructive Surgery at University of Louisville in Louisville, KY. A rhomboid flap, a bilobed flap, or a rotational flap was utilized. Table 1 delineates patient demographics and characteristics.

## CASE 1: RHOMBOID FLAP

**Figure 1:**
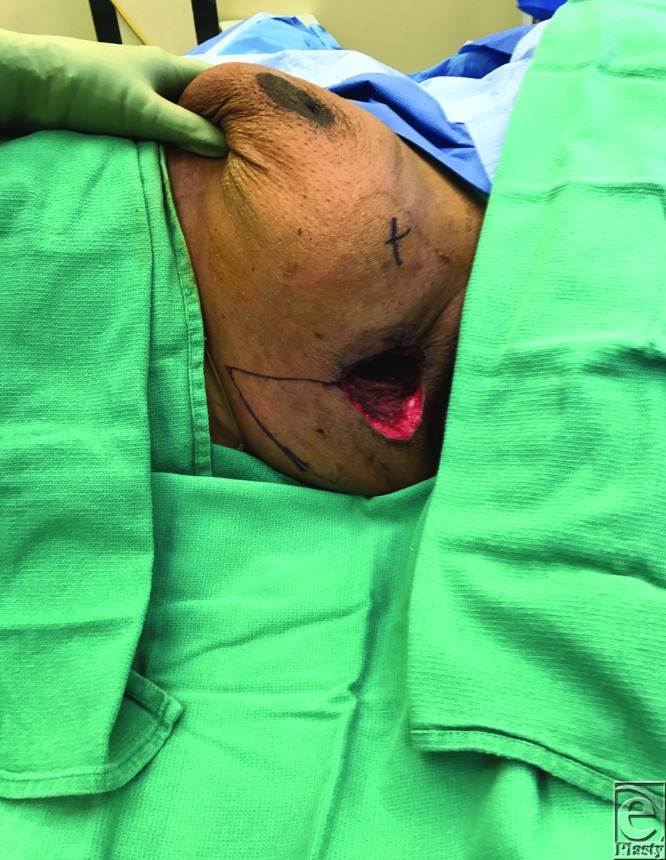
Defect postexcision with prospective rhomboid flap design.

**Figure 2:**
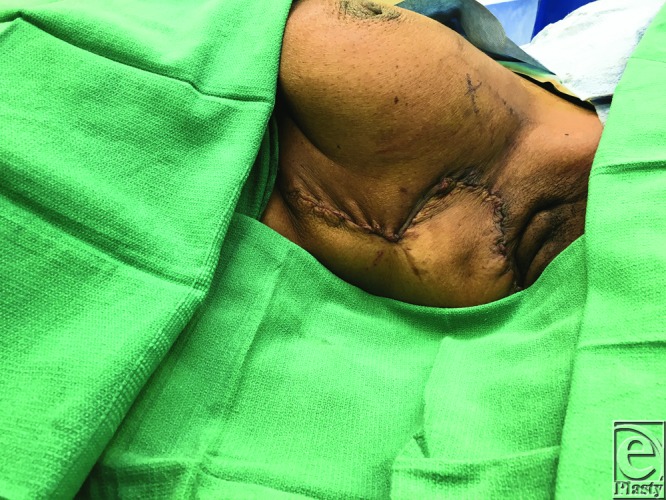
Closure with rhomboid local flap.

A 47-year-old African American woman underwent excision of a DFSP (dermofibrosarcoma protuberans) of her superolateral breast, resulting in a defect that measured 10 × 10 cm. An inferiorly based fasciocutaneous rhomboid flap 10 × 10 cm in size was raised and transposed to fill this defect. The flap was inset over a 19Fr Blake drain. Figure 1 demonstrates the design of the flap and defect, and Figure 2 demonstrates post–flap inset.

## CASES 2 to 4: BILOBED FLAPS

**Figure 3:**
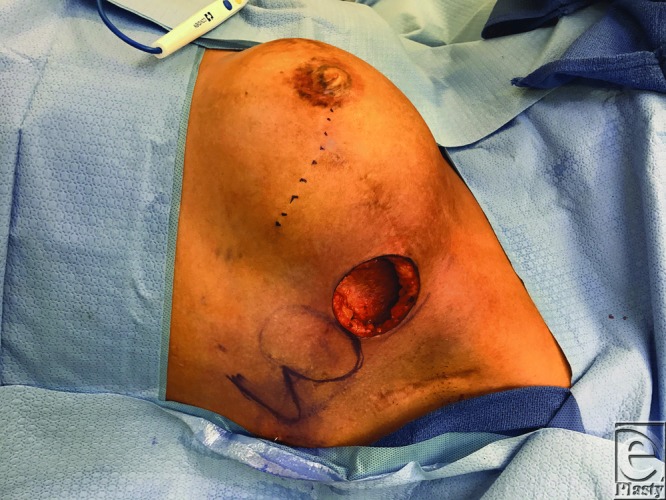
Defect with prospective design of bilobed flap (case 3).

**Figure 4:**
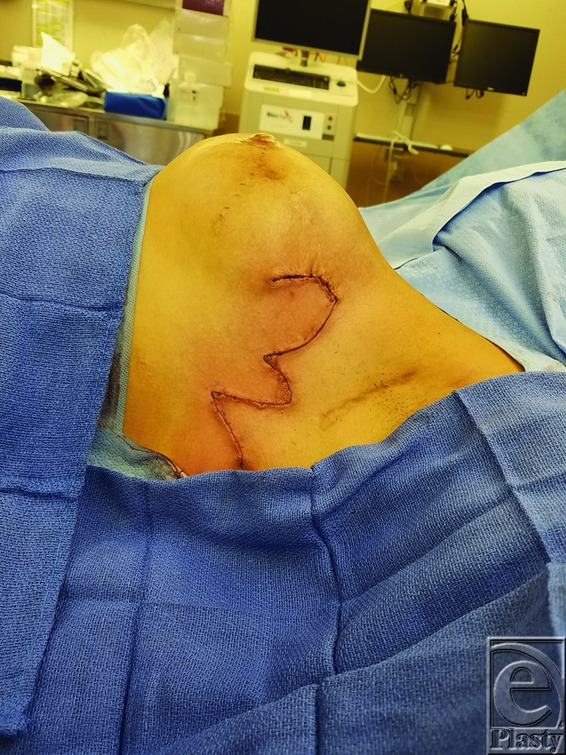
Closure with bilobed flap (case 3).

Fasciocutaneous bilobed flaps were created using adjacent tissue in each of the 3 cases. Highlighted later is case 3. The flap was raised at the level of fascia, elevated, and inset into the wound over a size 19Fr Blake drain. Figure 3 demonstrates the design of the flap and defect, and Figure 4 demonstrates post–flap inset.

## CASE 5: ROTATIONAL FLAP

**Figure 5:**
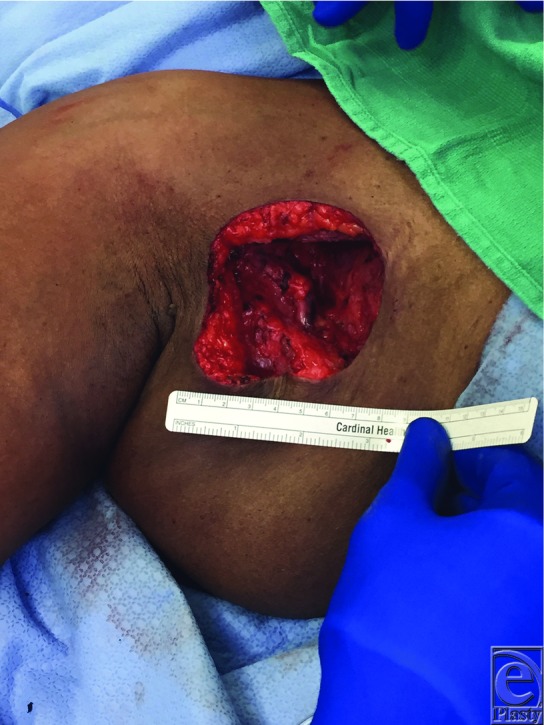
Defect after excision of recurrent invasive ductal carcinoma.

**Figure 6:**
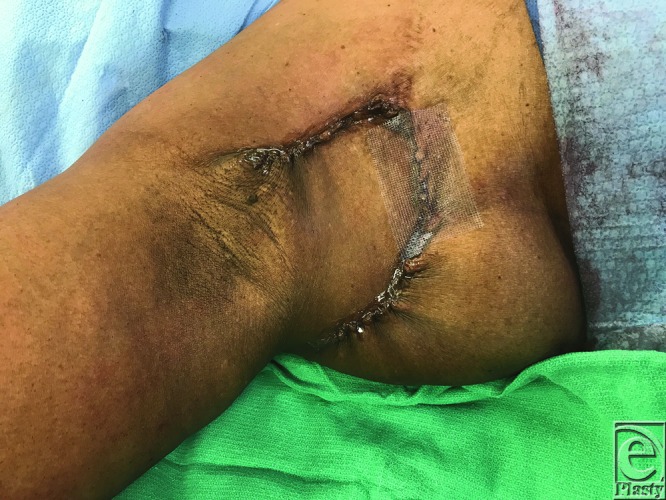
Closure with rotational flap.

A 64-year-old African American woman underwent excision for recurrent invasive ductal carcinoma in her lateral right breast/axillary region. The resulting defect was 8 × 8 cm. Given ample axillary laxity, the decision was made to perform a fasciocutaneous rotational flap for reconstruction of the defect. Figure 5 demonstrates the design of the flap and defect, and Figure 6 demonstrates post–flap inset.

## RESULTS

All patients underwent reconstruction of lateral breast defects with RPFs. There was no incidence of seroma or wound infection in any of the 5 women. Patient 4 developed a large hematoma postoperatively that necessitated rehospitalization and surgical evacuation; of note, her cardiac history necessitated therapeutic warfarin and enoxaparin pre- and postoperatively. All flaps except for case 2 were completely viable and healed well. The bilobed flap performed in case 2 experienced tip necrosis of distal 10% of the flap; this wound was also slow to heal, requiring weekly follow-up for surveillance and dressing changes. Patients overall were pleased with the cosmesis of the reconstruction. There were no cases of recurrence in any patient at the time of last follow-up. Summary of the results can be found in Table 2.

## DISCUSSION

Random patterned flaps are frequently used to reconstruct various defects of the head and neck^5^ but are seldom used in breast reconstruction.^1^^,^^2^ In small defects of the lateral breast or superior pole, RPFs offer an ideal reconstructive solution, yielding excellent outcomes and minimal morbidity.^1^^,^^3^^,^^4^ We describe 3 varieties of RPF utilized in 5 women with lateral breast defects of various causes.

Amongst flap selection, the choices range in decreasing complexity from free flaps to locoregional myocutaneous flaps to local flaps, which are further divided into perforator flaps and RPFs, as used in our patient population.^6^ Regarding local flaps, a delineation in classification is made by the arterial supply, where RPFs are supplied by dermal and subdermal plexuses, and pedicled flaps incorporate anatomically distinct vasculature along the long axis of the flap.^7^ The vasculature is rich and redundant in the breast and axilla, composed of several branches of the axillary artery.^8^ This correlates to robust dermal-subdermal plexuses that vascularize the RPF from this area.^5^^,^^7^^,^^8^ Kubo et al^9^ noted viability with rhomboid flaps up to sizes of 20 × 20 cm. Another factor guiding flap selection is location of tissue excess. Random patterned flaps can be harvested using the pinch technique to orient the donor area along a relaxed skin tension line.^10^^,^^11^ The subaxillary region provides ample tissue in most patients that can be transposed and inset into the defect site.^3^ Several flap designs should be considered that best utilize this adjacent tissue to best serve the patient.^12^ We offer 3 prospective designs: a rhomboid, a bilobed, and a rotational flap.

First described by Limberg,^10^^,^^13^ the rhomboid flap is a transposition flap in the shape of a parallelogram with two 120° angles and two 60° angles. Eight total rhomboid flaps can be conceived for any defect^10^; thus, it is of little surprise that this flap has found application in several parts of the body,^14^ including the breast.^9^^,^^15^ Advantages of the rhomboid flap include technical simplicity relying on easily taught geometry,^16^ coverage of large defects with good viability,^9^^,^^16^ and excellent match of tissue.^16^ Kubo et al^9^ have also noted decreased length of surgery and hospital stay with rhomboid flap breast reconstruction. Drawbacks include increased tension with lack of laxity in adjacent tissue, which generally is not a problem in most women in the subaxillary region,^3^ and the “zig-zag” scars, which although cannot be hidden entirely in tension lines,^16^ can be sequestered in the axillary region as we have done.

The bilobed flap is a transposition flap that was first described by Esser in 1918 and has a primary application in the closure of nasal defects.^17^^,^^18^ Others have expanded its utilization beyond this application to include truncal, extremity, and other facial regions.^18^^-^20 To the best of our knowledge, application of this flap in the setting of breast defects has remained undescribed. The bilobed flap allows for greater utilization of tissue versus other transposition flaps and can minimize tension across a wound as compared with a single lobed transposition flap.^18^ This is particularly suitable in areas where a single lobed transposition flap would result in excess tension across the wound.^21^ These advantages were important in case 3, where limited axillary tissue was available for transposition. Disadvantages include a more complicated design and inability to camouflage the curvilinear scars within tension lines.^21^

The rotational flap is another RPF that has been broadly applied to reconstruct defects throughout the body and has proved useful for small to moderate breast defects of multiple regions of the breast.^2^^,^^22^^,^^23^ The essence of this flap is a semicircle pivoted into a triangular defect; a circular defect necessitates being converted into a triangular one for use of this flap.^24^^,^^25^ Advantages of the rotational flap include flexibility resulting in greater utility for medial breast defects^22^^,^^23^ and a broad base providing robust vascularity allowing applicability to smokers and patients with vascular disease,^21^^,^^26^ minimal disruption to local lymphatics, vasculature, and nerves,^21^ and a design that allows for incisions to be well camouflaged.^26^ Disadvantages include potential need for dog-ear correction,^26^ rare necessity for skin graft closure of secondary defect, and potentially high degrees of tension within the flap impairing distal perfusion.^12^

While we highlight the usefulness of RPFs for breast reconstruction, they do not replace pedicled flaps and myocutaneous flaps for larger defects.^6^ The limited amount of adjacent tissue would not sufficiently cover these large defects^3^ and there would be concern of viability with the random nature of vascular supply, although in this series we demonstrated viability with flaps up to 10 × 10 cm. The location of the defect also impacts the choice of reconstruction; some suggest that the lower pole is more amenable to reduction techniques, while the superior pole and lateral region are amenable to RPF reconstruction.^3^ Others have stated that RPF reconstruction is possible for inner quadrant and lower pole defects.^22^^,^^23^ Our case series included only lateral defects, limiting applicability of our findings to reconstruction at other sites of the breast. Another important limitation is the size of this study, which limits overall generalizability.

We conclude that RPFs are a useful, albeit infrequently used, tool in the armamentarium of oncoplastic breast surgery techniques. They offer a technically simple and swift reconstructive solution that can yield excellent results. Multiple flaps can be designed to best utilize the adjacent tissue. Lateral breast defects in patients with ample axillary laxity are particularly amenable to reconstruction by this means.

## Figures and Tables

**Table 1:** Demographics and patient characteristics

Case	1	2	3	4	5
Age, y	47	84	33	89	64
Body mass index, kg/m^2^	22.6	22.5	27.8	27.6	37.6
Pathology	Dermofibrosarcoma protuberans	Basal cell carcinoma	Invasive ductal carcinoma	Squamous cell carcinoma	Invasive ductal carcinoma
Defect size, cm	10 × 10	5 × 6	5 × 5	9.5 × 9.5	8 × 8
Laterality	Left	Right	Left	Right	Right
Location	Superolateral	Lateral	Superolateral	Lateral	Lateral
Flap selection	Rhomboid	Bilobed	Bilobed	Bilobed	Rotational
Comorbidities	Multiple sclerosis	Type II diabetes mellitus, peripheral vascular disease	Former smoker	Heart failure, aortic valve replacement (taking warfarin)	Type II diabetes mellitus, stage III chronic kidney disease

**Table 2:** Postoperative results

Case	1	2	3	4	5
Age, y	47	84	33	89	64
Flap complication	None	Tip necrosis	None	Hematoma	None
Postresection radiotherapy	Yes	No	Yes	No	Yes
